# Optimal location of logistics distribution centres with swarm intelligent clustering algorithms

**DOI:** 10.1371/journal.pone.0271928

**Published:** 2022-08-25

**Authors:** Tsung-Xian Lin, Zhong-huan Wu, Wen-Tsao Pan

**Affiliations:** 1 Department of Management, Guangzhou Huashang College, Guangzhou, China; 2 Institute for Economic and Social Research, Guangzhou Huashang College, Guangzhou, China; 3 School of business, Guangdong University of Foreign Studies, Guangzhou, China; Torrens University Australia, AUSTRALIA

## Abstract

A clustering algorithm is a solution for grouping a set of objects and for distribution centre location problems. But the common K-means clustering algorithm may give local optimal solutions. Swarm intelligent algorithms simulate the social behaviours of animals and avoid local optimal solutions. We employ three swarm intelligent algorithms to avoid these solutions. We propose a new algorithm for the clustering problem, the fruit-fly optimization K-means algorithm (FOA K-means). We designed a distribution centre location problem and three clustering indicators to evaluate the performance of algorithms. We compare the algorithms of K-means with the ant colony optimization algorithm (ACO K-means), particle swarm optimization algorithm (PSO K-means), and fruit-fly optimization algorithm. We find K-Means modified by the fruit-fly optimization algorithm (FOA K-means) has the best performance on convergence speed and three clustering indicators, compactness, separation, and integration. Thus, we can apply FOA K-means to improve the distribution centre location solution and the efficiency for distribution in the future.

## 1 Introduction

The development of Machine Learning provides supervised learning and unsupervised learning algorithms. Supervised learning uses labelled data sets to train algorithms to classify data and predict outcomes. We can use a linear model to make an economic data forecast, like the influence of educational input on gross national income (GNI) [1]. A deep neural network is a widely used non-linear model for animal and human face recognition [2] and stock returns and options market forecast [3–5]. Unlike supervised learning, unsupervised learning can classify unlabelled data. The clustering algorithm is an unsupervised learning method that groups a set of unlabelled objects.

The clustering algorithm can help solve many problems, like large databases and customer segmentation. Many researchers developed clustering algorithms for various customers segmentation, like in the automobile retailer industry [6], airline customers [7], e-commerce companies [8,9]. Clustering can help forecast customer demand and improve marketing strategy for companies [10]. There have been many clustering methods. The K-means clustering algorithm is widely used in previous cases, and k-means clustering has been widely studied with various extensions in the literature and applied in many substantive areas [11–14]. However, these k-means clustering algorithms are usually initialized and need to be given some cluster priors. In general, the cluster number is unknown. In this case, the validity index can be used to find the cluster numbers where they should be independent of the clustering algorithm [15]. Many clustering effectiveness metrics for k-means clustering algorithms are already in the literature, such as Bayesian Information Criterion (BIC) [16], Akaike Information Criterion (AIC) [17], Dunn Index [18], Silhouette Width (SW) [19], Calinski and Harabasz Index (CH) [20], Gap Statistics [21], Generalized Dunn Index (DNg) [22], and modified Dunn indices (DNs) [23].so we focus on the K-means clustering algorithm in this paper [24].

The K-means clustering algorithm is efficient, but the simple design may suffer from local optimal solutions. Clustering is combinations of data points in different clusters and, thus, is a combinatorial optimization problem. In practice it has been found that metaheuristic algorithms like Genetic Algorithms (GAs) are better choice for combinatorial optimization [25]. Some researchers try the hybrid genetic algorithm to improve K-means [26,27]. We try to develop K-Means with a swarm intelligence algorithm because the swarm intelligence algorithms have advantages to find a better solution in non-convex programming problems and the swarm intelligence algorithms have advantages to find a better solution in non-convex programming problems compared with other algorithms. Particle swarm optimization (PSO) [28] and Ant Colony Optimization (ACO) [29] have good features to simulate animals behaviours to avoid local solutions. PSO is a new biological evolution algorithm. It starts from a random solution, finds the optimal solution through iteration, and evaluates the quality of the optimal solution through fitness. It is simpler than the genetic algorithm, and there is no "crossover" and " mutation " compared with genetic algorithm, which finds the global optimum by searching optimum value. Each in the ACO algorithm can change the surrounding environment by releasing pheromones, and each can perceive the real-time changes in the surrounding environment, and the individuals communicate indirectly. At the same time, the distributed computing method is adopted in the search process, and multiple individuals perform parallel computing, which greatly improves the computing ability and operating efficiency. We applied the clustering algorithm modified by PSO [30] and ACO [31]. Additionally, we develop a new swarm intelligence algorithm, Fruit fly Optimization (FOA), to the clustering algorithm. Fruit fly Optimization has been proven to find a satisfactory solution and good convergence speed [32–34]. By simulating the predation process of fruit flies using their keen sense of smell and vision, FOA realizes a group iterative search. The principle of FOA is easy to understand, simple to operate, easy to implement, and has strong local search ability. The three algorithms probability search method is not easy to fall into the local optimum, and it is easy to find the global optimum solution. Given the advantage of K-Means and FOA, we can obtain a good algorithm for the clustering problem.

Logistics distribution centres are intensive and specialized, serving a set or region of customers. The logistics distribution centres store, sort, distribute, and deliver goods based on customer requirements. Selecting algorithms to achieve lower costs will help companies work efficiently and create more value for society [35]. There are many positive cases about location, such as e-commerce in a city [36], the clothing industry [37], and the locations for relief distribution and victim evacuation after a disaster [38]. Clustering and location problems have similar objectives, so we use a clustering algorithm to help cluster customers and locate the distribution centres.

This paper is organized as follows: Section 2 Methodology shows the details of common K-Means clustering, ACO, PSO, and FOA K-means. Section 3 Results and discussion conducts empirical analysis and describes the superior performance of FOA K-means. Section 4 presents the conclusions.

## 2 Methodology

The K-means algorithm is an efficient and widely used algorithm for clustering. The simple design suffers from local optimal solutions in many cases. But the swarm intelligence algorithms can find better solutions in non-convex programming problems. Three swarm intelligence algorithms are employed to solve the location problem for distribution problems. The ant colony optimization algorithm, particle swarm optimization algorithm, and fruit-fly optimization algorithm are introduced as follows.

### 2.1 K-Means algorithm

K-Means clustering is a fast, robust, and simple algorithm with an effective convergence property that gives results when data sets are distinctly separated from each other. It performs well when the number of cluster centres is specified by a well-defined list of types shown in the data. The process of common K-Means is:

Step 1: Randomly select K objects from N objects as the cluster centre, where K is the required target classification number.
Step 2: Calculate the distance of all remaining objects to each cluster centre. Use the Euclidean distance to assign objects to the same categories and nearest cluster centre.
Step 3: Determine the cluster centres based on the mean properties of each object in the same categories.
Step 4: Repeat steps (2) and (3) until the new cluster centre is equal to or less than the target threshold and the clustering algorithm ends.

### 2.2 Ant colony optimization algorithm

The ant colony optimization algorithm (ACO) is a probabilistic algorithm for route optimization and clustering analysis. This application performs well for transportation and telecommunication networks [31]. The ant colony algorithm was proposed by Marco Dorigo in 1992 and shows how ants find their way to food. The main characteristics of this algorithm include distributed computing, heuristic search, and feedback. It is an effective algorithm to find a satisfactory solution and avoid falling into local solutions.

**Fig 1:**
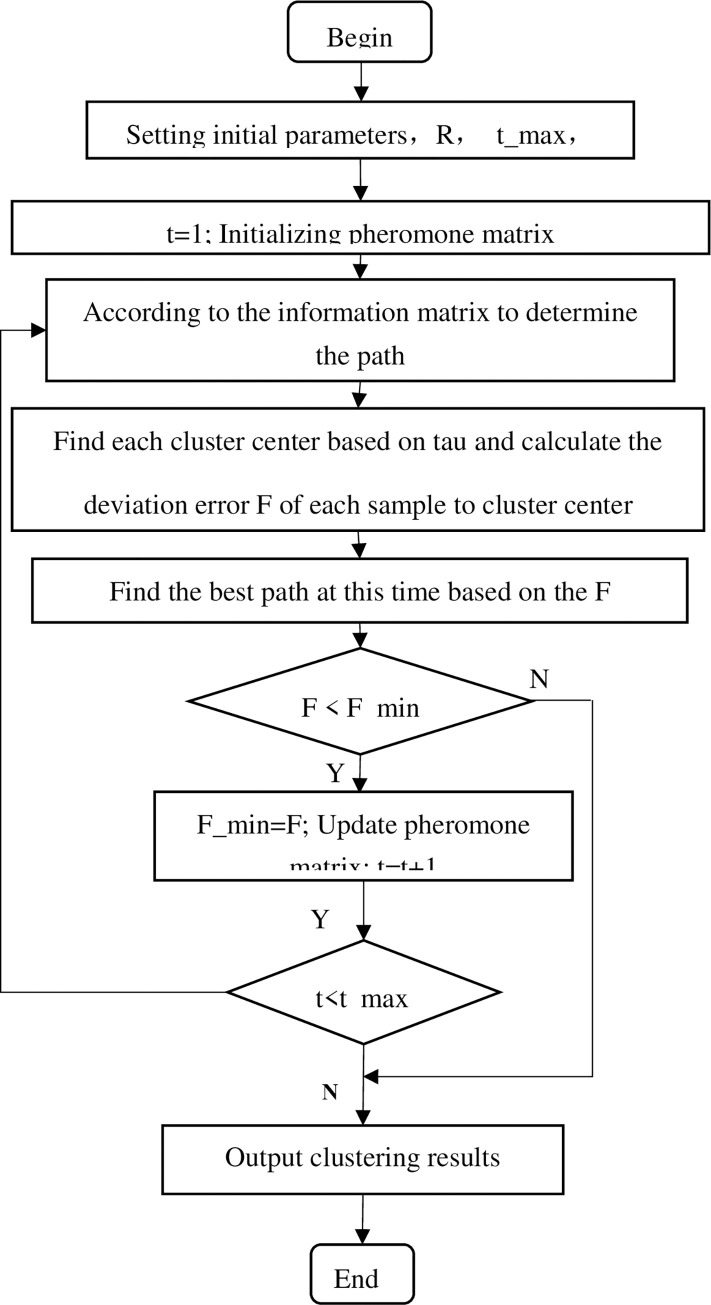
Flowchart of ant colony optimization algorithm.

The clustering algorithm modified by the ant colony algorithm appears in Fig 1. The process is as follows:

Step 1: Initialize the ant colony parameters. Set the maximum number of iterations to 1000. Set the number of cluster samples, attributes, and cluster centres. Set the pheromone evaporation rate rho = 0.1, which will update the pheromone matrix. Set the number R of ants (the solutions) to 100.
Step 2: Initialize the pheromone matrix. The pheromone matrix is a matrix of N*K (sample number * cluster number). Set all pheromone initial values to 0.01.
Step 3: All ants construct a solution set according to the pheromone matrix tau_N×K_. The higher tau_i,k_, object i is assigned to categories k and centre k.
Step 4: Generate the vector r_1×N_ for each ant. If the corresponding value r_i_ is less than the set pheromone threshold q = 0.9, the path takes the maximum pheromone. If there are multiple maximum values, randomly take one of them from the same maximum value as the path. If the pheromone is greater than the set threshold q = 0.9, the ratio of each path pheromone to the total pheromone of the sample is obtained. The initial path is determined by probabilities based on the pheromone proportion of each path. In this step, each ant may have a different solution.
Step 5: Determine the new centres based on the mean attributes of objects in the same categories.
Step 6: The deviation error F_ij_ is the Euclidean distances of each object to the cluster centre j. The smaller the F_ij_ is, the better the clustering effect is. Calculate the sum D_i_ of deviation smallest error F_*ij*_ for each sample i to the nearest cluster centre j in each ant and find the best ant solutions D_best_.
Step 7: Update the pheromone value according to the best L solutions (L = 2) in the last step. The pheromone matrix update is as follows:

$${tau}_{i,j\;in\;bestsolution}=(1-rho)\times {tau}_{i,j\;in\;bestsolution}+1/F$$

$$F=\sum_{i=1}^{L}D_{i\;of\;smallest}$$

tau_*i*,*j in bestsolution*_ is updated according to the best solution.

Step 8: Determine whether the maximum number of iterations is reached. If not reached, return step (3); if reached, the optimal cluster solution is output.

### 2.3 Particle swarm optimization algorithm

Kennedy and Eberhart first introduced the particle swarm optimization algorithm (PSO). PSO is effective and highly efficient as well [39]. This algorithm simulates the social behaviours of birds and is a good optimization algorithm with relatively low iteration numbers. A study finds that adding environmental factors can improve the performance of PSO in searching strategy, but the question is how to select the environmental factor more effectively [40].

In the PSO algorithm, each particle can remember the best solution that it has searched for. P_id_ is the best position that the entire particle swarm has experienced. P_gd_ is the best solution in the current search. Each particle has a speed, V represents the velocity of the i-th particle in the d-th dimension, w is the inertia weight, η_1_, η_2_ are the importance parameters for P_id_ and P_gd_, and rand() is the random function that generates a floating variable in the range [0,1]. Calculate:

$$V_{id}={wV}_{id}+\eta_{1}rand()(P_{id}-X_{id})+\eta_{2}rand()(P_{gd}-X_{id}),\;\eta_{1}=\eta_{2}=1.2$$ (1)

$$w=w_{max}-t*(w_{max}-w_{min})/M,w_{max}=0.9,w_{min}=0.4,M∼iterations$$ (2)

Then the updated position of particle is:

$$X_{id}=X_{id}+V_{id}$$ (3)

The process of the PSO K-Means Algorithm is as follows:

Step 1: Initialize the particles and randomly assign each object to a category. Determine cluster centres based on the mean of the objects in the same category. Initialize the velocity of the particles, iterations, and group size.
Step 2: For each particle, calculate the fitness value, and compare it to the recorded best fitness value of P_id_. If there is one better than P_id_, update P_id_;
Step 3: Compare each particle fitness value with the best position in the current group P_gd_. If there is a better one, update P_gd_;
Step 4: Determine the velocity and position of the particles based on Formula (1) and Formula (2).
Step 5: Apply the K-means optimization. Update the cluster centre, apply the nearest neighbour rule, and update the particle.
Step 6: If the end condition is reached (maximum iterations M = 200 in this case), the process ends. Otherwise, return to step (2).

### 2.4 Fruit fly optimization algorithm

The fruit fly optimization algorithm (FOA) was proposed by Professor Pan (2011) and has been widely applied in solving programming problems. These problems include logistics storage selection [22] and a mutual fund forecasting model [41], which optimize the parameters of a general regression neural network in the forecasting model and obtain a better solution than PSO. The fruit-fly optimization algorithm simulates the fruit-fly behaviours of seeking food and contributes a swarm intelligence optimization algorithm. The process of the standard fruit fly algorithm is as follows:

Step 1: Initialize the population size Sizepop, the number of iterations Maxgen, and the random location of the fruit fly.
Step 2: Each fruit fly randomly searches for food in all directions.
Step 3: Calculate the distance Dist_*i*_ from each fruit fly to the origin and set the smell value *S*_*i*_.

$$\begin{array}{l} {\mathrm{Dist}}_{i}=\sqrt{X_{i}^{2}+Y_{i}^{2}}\\ S_{i}=\frac{1}{{\mathrm{Dist}}_{i}} \end{array}$$

Step 4: Substitute the obtained smell value to the fitness function and determine the best fruit fly.
Step 5: Identify the best-performing fruit flies in the fruit fly population.
Step 6: Record the optimal taste density value and the specific X, Y coordinates.

$$\begin{array}{l} \;{\mathrm{Smell}}_{i}=Function(S_{i})\\ \left[bestSmell,\;bestIndex]=max({\mathrm{Smell}}_{i}\right) \end{array}$$

$$\begin{array}{l} \;Smellbest=best\;Smell\\ X_{\_\mathrm{axis}}=X(bestIndex)\\ Y_{\_\mathrm{axis}}=Y(bestIndex) \end{array}$$

Step 7: Start the iteration, repeat (2) through (6), and if the current iteration time is less than maximum iterations Maxgen, then perform step (2). Otherwise, end the calculation.

The Basic FOA will be reconstructed to make it more suitable for solving clustering problems based on the K-Means algorithm.

### 2.5 K-Means modified by FOA

In the previous result, we can see some flaws while we are using the other algorithms. ACO avoids falling into a local optimal solution, but the convergence speed is low., On the other hand, PSO updates the decisive variables well, but compared with K-Means, it does not show its advantage of being a swarm intelligent algorithm. So, we want to develop an algorithm to combine the characteristics of good convergence speed of K-Means and FOA and Modified FOA. The K-Means algorithm is the new algorithm to satisfy our demands.

We develop the modified FOA K-Means algorithm as follows:

Step 1: Design the fruit fly structure for clustering. Each fruit fly is a cluster centre. There are many objects, assigned to N categories. There are N centres, and each centre has k attributes. X_i_, Y_i_ represent the coordinate vectors of the i-th fruit fly. The size of the fruit-fly population is sizepop. When initializing, x_i_, y_i_ are randomly generated, and rands(1, N×k) represents a random number of (0,1), which produces one row and N×k columns. *rcc* is the max attribute value. In the following case, we set it to 6000. D_*i*_ is the distance from each fruit fly to the origin.

$$x_{i},y_{i}=rand*rcc$$

rcc=maxattribute

$$X_{i}=\{x_{1},x_{2},x_{3},\ldots ,x_{N\times k}\}$$

$$Y_{i}=\{y_{1},y_{2},y_{3},\ldots ,y_{N\times k}\}$$

$$D_{i}=\sqrt{{X_{i}}^{2}+{Y_{i}}^{2}}+Z_{i,modi}(when\;initializing\;vector\;Z_{i,modi}=0)$$

Step 2: The objects closest to the same cluster centre are categorized into the same class. Sum up the Euclidean distances from the cluster centre to each object. Apply the K-means method to generate a new cluster centre CentreBest with the shortest total distance of all solutions.
Step 3: Determine the adjust vector Z_i,modi_

$$Z_{i,modi}={CentreBest}_{i}-{\left({X_{i,axis}}^{2}+{Y_{i,axis}}^{2}\right)}^{1/2}$$

Step 4: Determine the variation from best fruit fly. Compare the original best fruit fly with other flies with mutations. The method of mutation is as follows.

$$X_{i}=X_{i,best\;axis}+(2\times rands(1,N\times k)-1)\times ssc$$

$$Y_{i}=Y_{i,best\;axis}+(2\times rands(1,N\times k)-1)\times ssc$$

$$ssc=\frac{\max\;attribute}{2}$$

Step 5: Determine whether max iterations is reached. If not, return to step 2. Otherwise, output the result. We set 200 iterations.

## 3 Results and discussion

To evaluate the performance of swarm intelligence algorithms, we conduct a calculation test case.

### 3.1 Location problem

**Fig 2:**
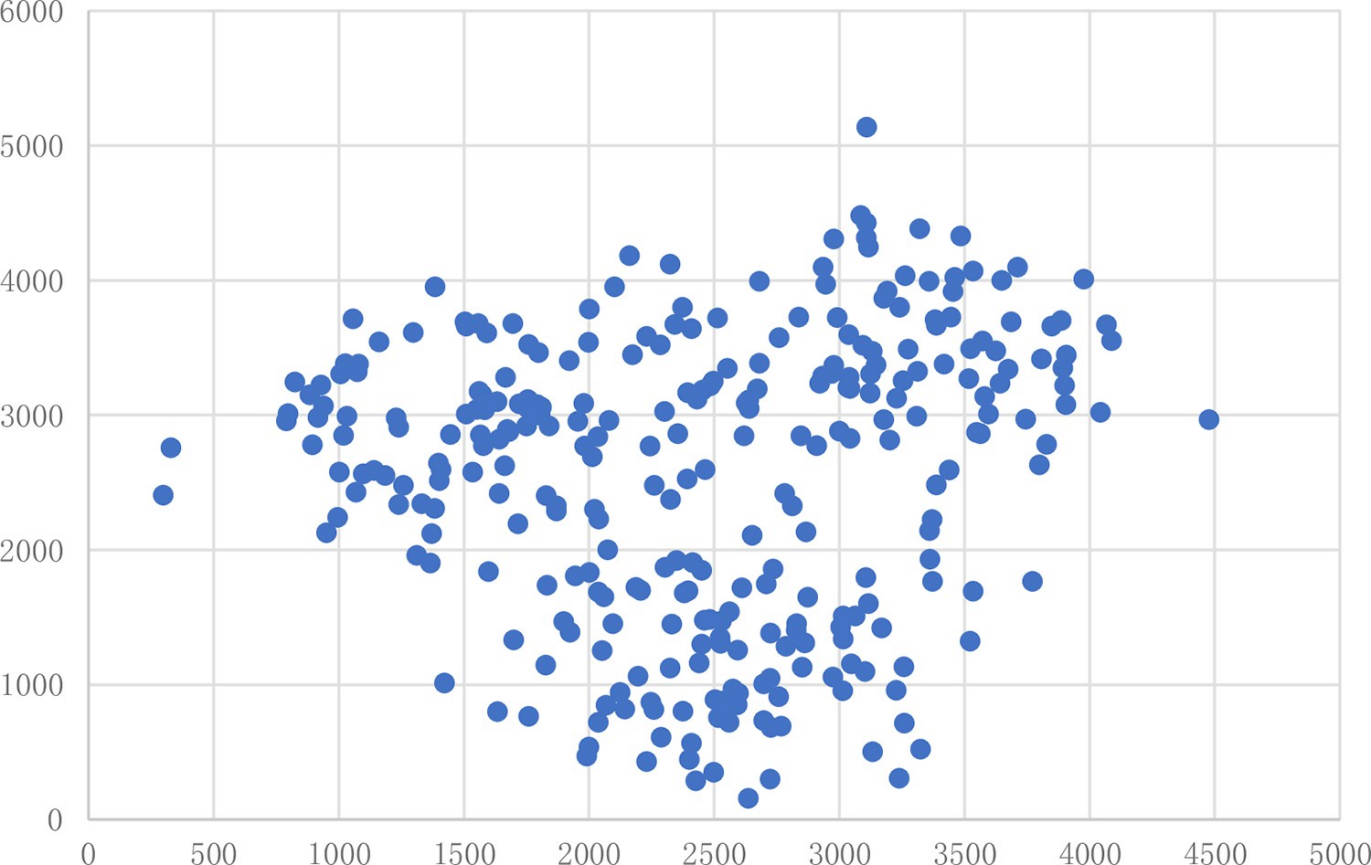
Retailers location map.

In a city, retailers need to send goods to many customers. They must build distribution centres to serve all customers. The locations of customers appear in Fig 2.

**Fig 3:**
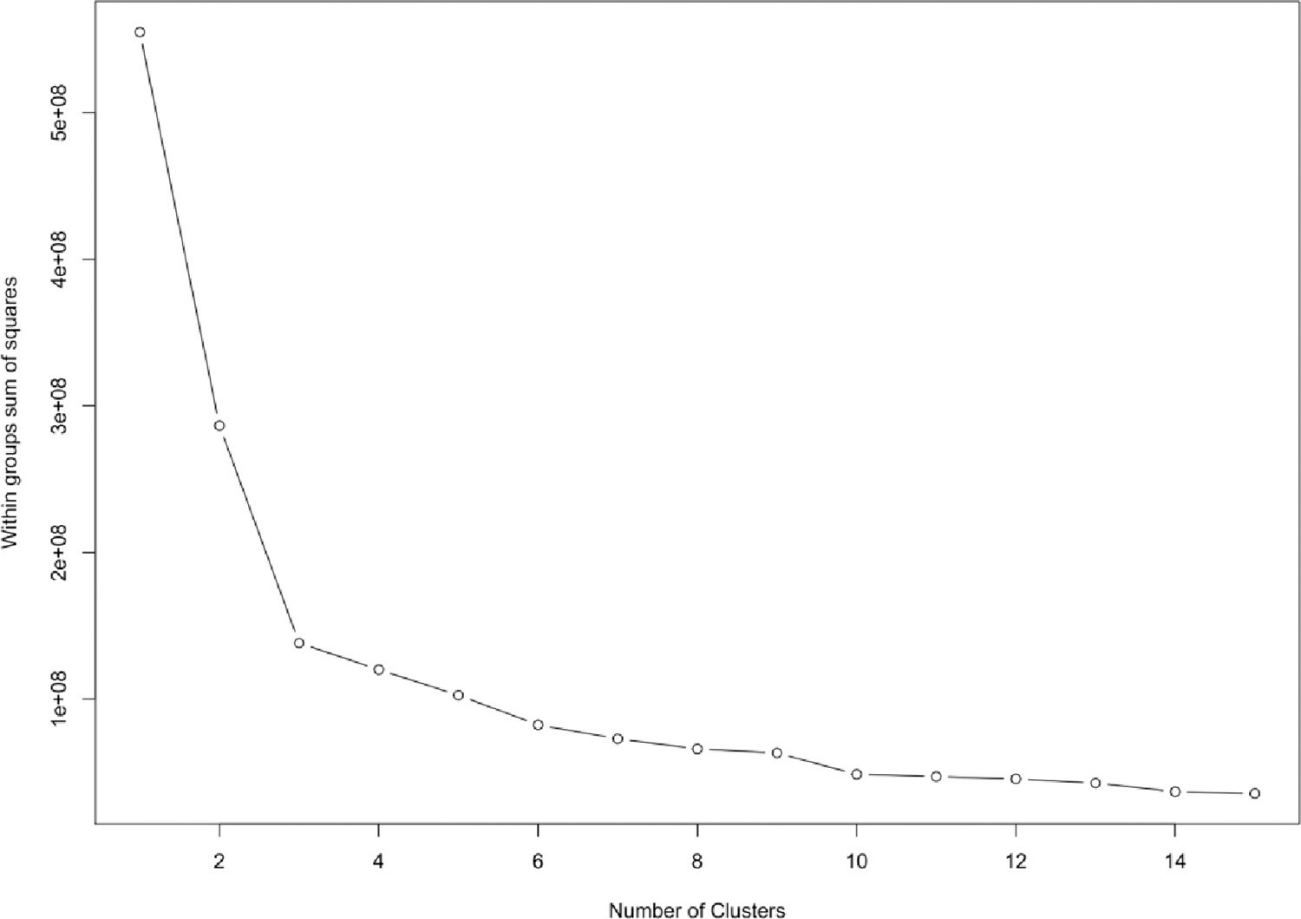
Sum of squared error of various clusters.

We need to determine the number of distribution centres. Within a group, the sum of squared error (SSE) is a common method to determine the optimal number of clusters. In Fig 3, the optimal number of clusters is three. With more categories or clusters, there is a shorter distance within the group’s sum of squared error. We focus on the change of slope. The decline from one to three categories is rapid and then decreases slowly, so to reduce the construction cost, the optimal number of clusters is three. To serve the needs of the retailers, three logistics distribution centres should be built.

### 3.2 Results of the algorithms

#### 3.2.1 Common K-Means

**Fig 4:**
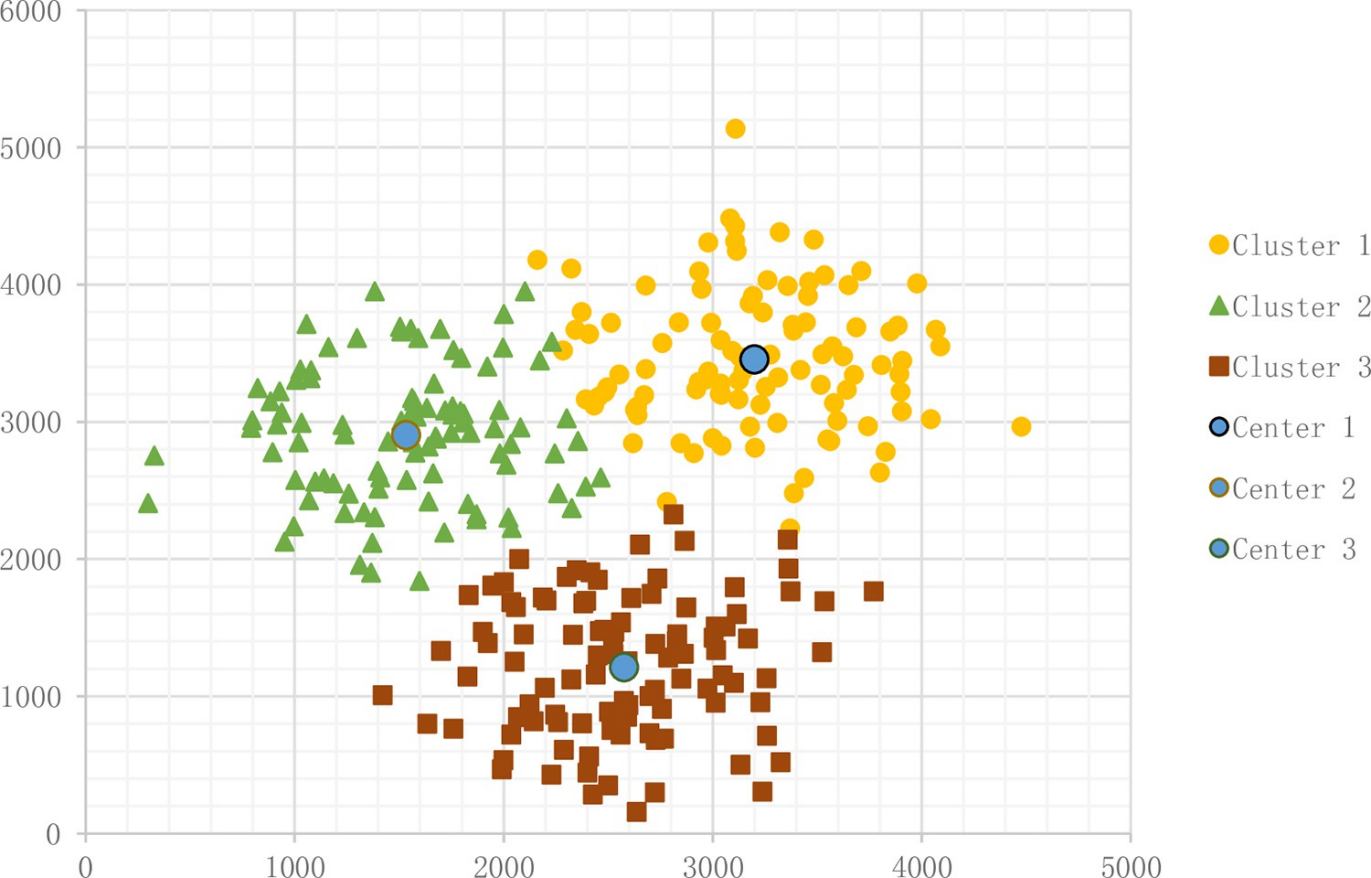
Result of common K-Means.

**Fig 5:**
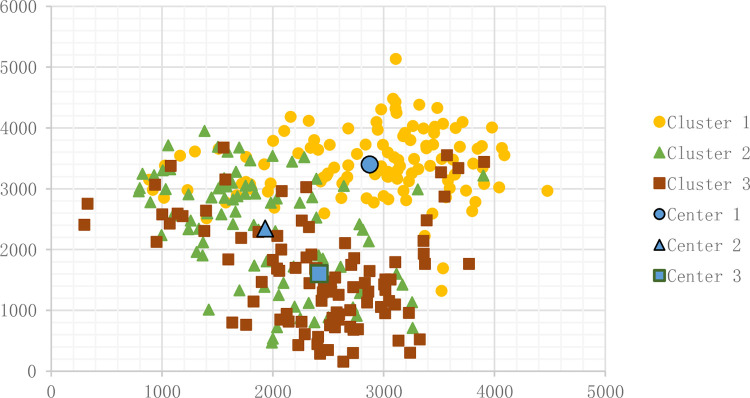
Result of ACO.

Means results appear in Fig 5. The sample number N is 300, and the cluster number K is 3. Fig 4 shows the result.

The distribution centres result appears in Table 1.

**Table 1:** K-Means distribution centres.

	Horizontal axis(X/m)	Vertical axis(Y/m)
Distribution centre 1	3198.532555	3458.119546
Distribution centre 2	1531.344733	2906.774243
Distribution centre 3	2570.173	1218.828

The total distance from each retailer to the distribution centres is 184400.41 m.

#### 3.2.2 K-Means modified by ACO

The ant colony algorithm is used to solve the problem. The initial parameters are as follows. The iteration is 200, the ant number is 200, the sample number is 300, and the cluster number is 3. After running ACO, we find that the ant colony clustering algorithm is unsuitable for this problem. The convergence is poor with more iterations. The result appears in Fig 5.

The distribution centres appear in Table 2:

**Table 2:** ACO distribution centres.

	Horizontal axis(X/m)	Vertical axis(Y/m)
Distribution Centre 1	2872.944	3401.305
Distribution Centre 2	1930.966	2349.514
Distribution Centre 3	2417.269	1607.756

The total distance from each retail customer to the distribution centres is 280379.72 m.

#### 3.2.3 K-Means modified by PSO

**Fig 6:**
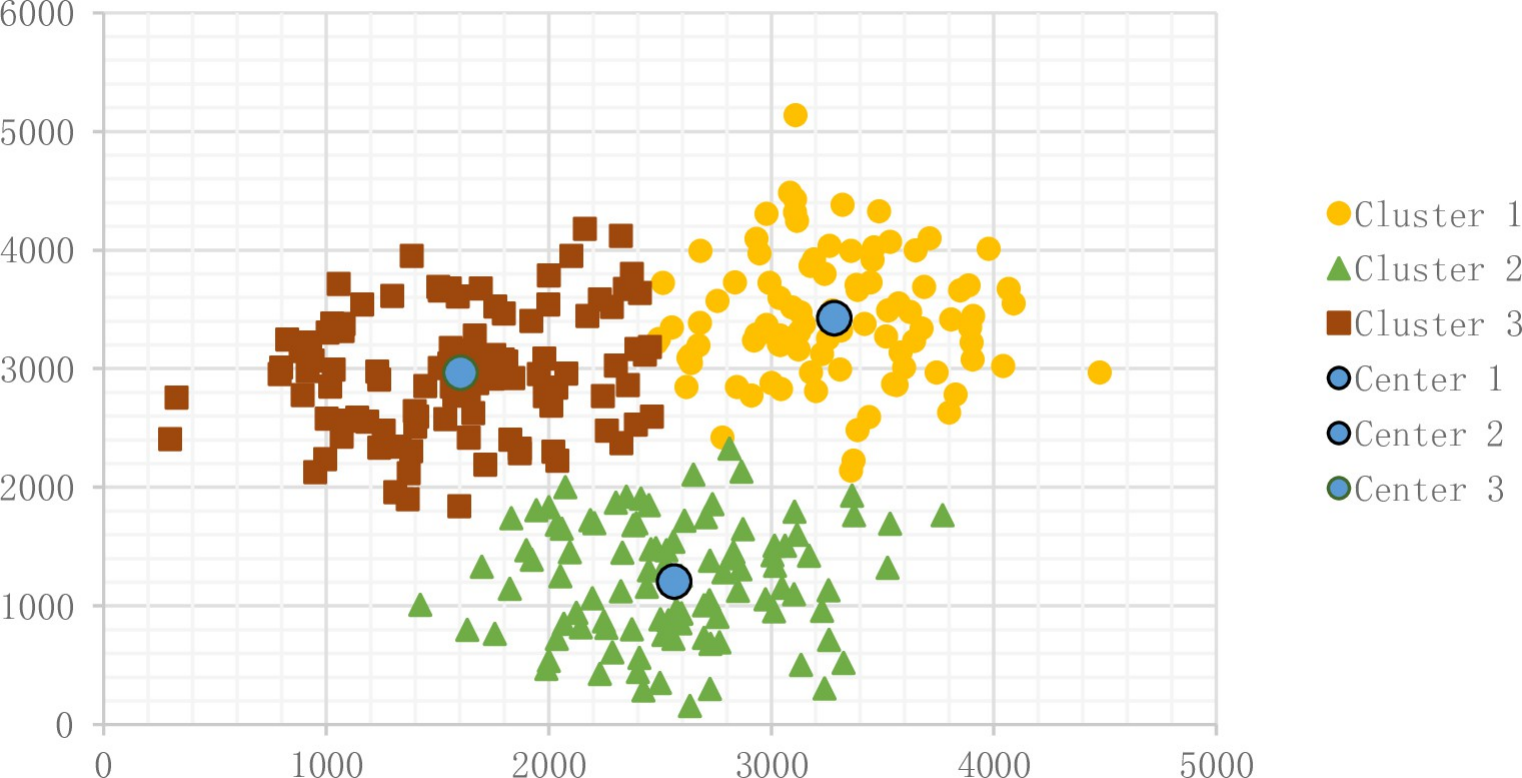
Result of PSO K-Means.

We set initial parameters: the iteration is 200, the particle size is 200, the sample number is 300, and the cluster number is 3. The result appears in Fig 6.

The distribution centres are shown in Table 3:

**Table 3:** PSO K-Means distribution centres.

	Horizontal axis(X/m)	Vertical axis(Y/m)
Distribution Centre 1	3282.131	3430.185
Distribution Centre 2	2562.433	1209.77
Distribution Centre 3	1601.736	2966.247

The total distance from each retailer to the distribution centres is 185227.7256 m.

#### 3.2.4 K-Means modified by FOA

In the previous result, we saw some flaws in the other algorithms. ACO avoids falling into local optimal solution, and PSO updates the decision variables but does not show its advantage of a swarm intelligent algorithm compared with K-Means. We develop a new FOA K-Means algorithm with good convergence speed and performance to avoid local optimal solutions.

**Fig 7:**
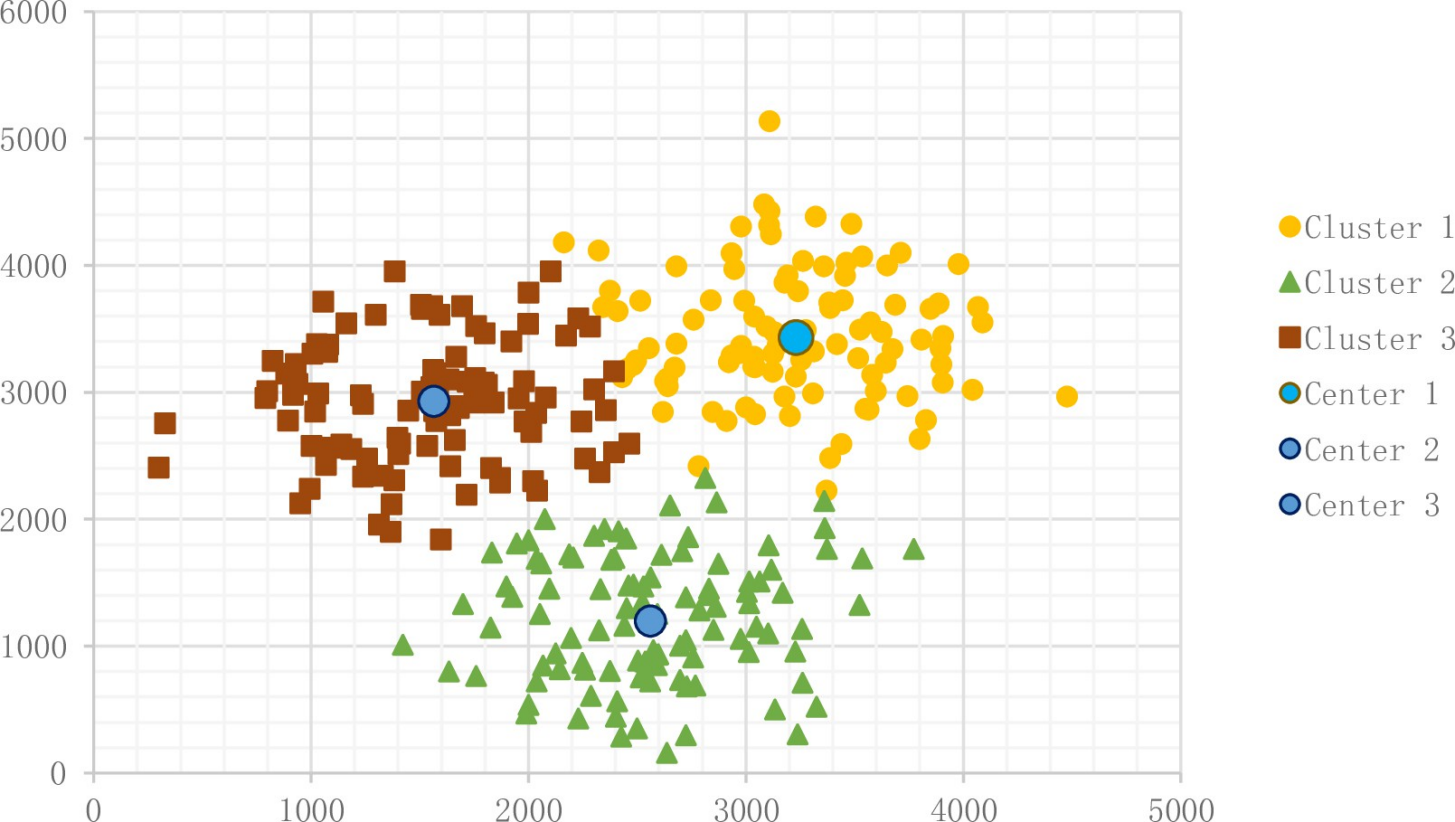
Result of MFOA K-Means.

The result of the fruit fly algorithm appears in Fig 7:

The distribution centres appear in Table 4:

**Table 4:** MFOA distribution centres.

	Horizontal axis(X/m)	Vertical axis(Y/m)
Distribution Centre 1	3231.8	3431.8
Distribution Centre 2	1562.7	2930.4
Distribution Centre 3	2559.9	1198.7

The total distance from each retailer to the distribution centres is 184365.38 m.

#### 3.2.5 Comparison of results

In Table 5, Total Distance is a compactness indicator, so smaller is better. Total Centre Distance is the separation indicator, so higher is better. Davies-Bouldin index is an integrated indicator, so smaller is better.

**Table 5:** Comparable indicators of the four algorithms.

Algorithms	Common K-Means	ACO	MFOA K-Means	PSO K-Means
Total Distance (m) *Compactness indicator	184400.41	280379.72	184365.38	185227.73
Total Centre Distance (m) *Separation indicator	6063.77	4149.43	6073.07	6079.44
Davies-Bouldin Index *Integrated indicator	1.846841	4.532969	1.846223476	1.855038066

**Fig 8:**
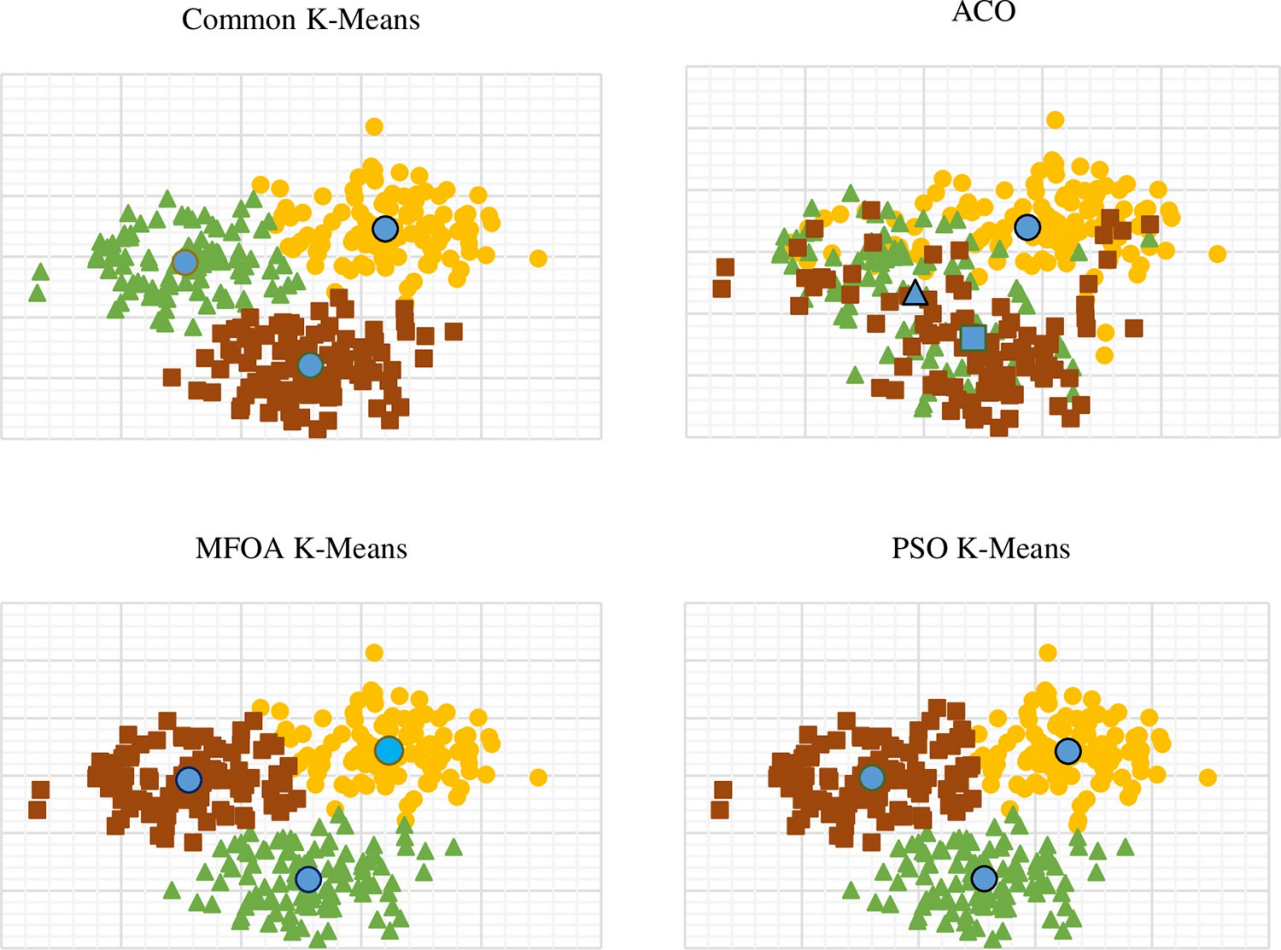
Comparison of the four results.

The comparison figure of the algorithms appears in Fig 8 and Table 5:

ACO is not suitable for cluster analysis in large clustering samples in the study due to the poor performance of three indicators. Total Distance is the highest, 280379.72. The total centre distance is the lowest, 4149.43. Davies-Bouldin Index is the highest, 4.53. Compared with Common K-Means, MFOA K-Means, and PSO K-Means, the convergence speed of ACO is obviously slower in the computational experiment.

The PSO K-Means and FOA K-Means algorithms can prevent the Common K-Means from falling into a local optimum as well. In this case, PSO K-Means does not perform better than common K-Means due to the higher compactness indicator and higher DBI. PSO K-Means is more likely to fall into a local optimal solution than FOA. The performance of FOA K-Means is better than that of common K-Means and has the best performance in three indicators. FOA K-Means has the smallest DBI, 1.846.

**Fig 9:**
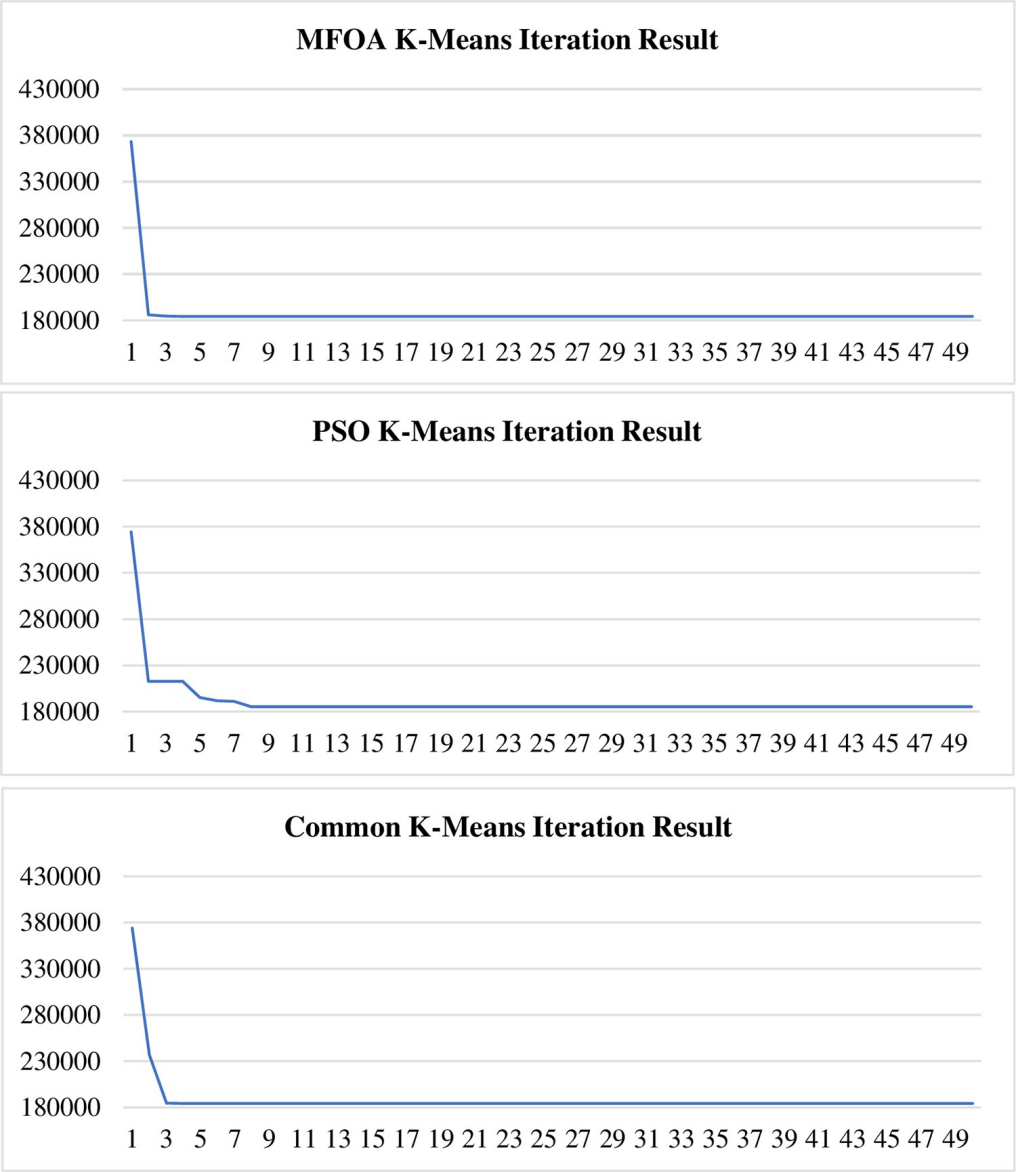
Three optimal clustering algorithms iteration result.

Through the results in Fig 9, we can also draw further conclusions:

In the results, we can see that when the FOA K-Means algorithm reached a local optimal state, it still could optimize the solution. If we run more tests on common K-Means, it might generate a worse solution than the previous result. FOA K-Means has a more stable performance. Another cluster intelligent algorithm, the particle swarm K-Means algorithm, compared to the modified fruit fly optimization K-Means algorithm, has little advantage in this problem.

## 4 Conclusions

We solved the location problem with swarm intelligent clustering algorithms (including the ant colony optimization algorithm, the fruit fly optimization K-Means algorithm, and the particle swarm optimization algorithm) and the common K-Means algorithm. We proposed a new swarm intelligent algorithm, the fruit fly optimization K-Means algorithm (FOA K-means). We compared the indicators of algorithm results. The FOA K-Means algorithm has the best performance in the DBI indicator, which has great significance for clustering problems in further studies. FOA K-means has the best performance on convergence speed and three clustering indicators, compactness, separation, and integration. ACO is not suitable for cluster analysis of the large clustering samples in the study due to the poor performance in three indicators. PSO K-Means is easier to fall into a local optimal solution than FOA. Therefore, FOA K-means is the best solution for distribution centre location problems and clustering.

The study has some deficiencies. To analyse the clustering problem further, some actual constraints are neglected in location problems. First, the location of the distribution centres is obtained using the mean of the coordinates. Second, the relative importance of different customers is not considered. Different customers have varios needs, which can affect the location of the distribution centres. The need for excellent customer service becomes important. And any business’ ability to understand each segment of its customers’ needs will give it a greater advantage in providing targeted customer service and developing customed centres. Future research can explore addressing these problems further.

## Supporting information

S1 Dataset (XLSX)
